# Supplementary material for: Selenium Nanomaterials Enhance the Nutrients and Functional Components of Fuding Dabai Tea
Source: Nanomaterials (Basel). 2024 Apr 15;14(8):681. doi: 10.3390/nano14080681 (PMC11053761; doi:10.3390/nano14080681)
Supplement: Supplementary file 1 [file nanomaterials-14-00681-s001.zip › nanomaterials-2934022-supplementary.pdf]

Selenium Nanomaterials Enhance the Nutrients and Functional Components of Fuding Dabai Tea

Xiaoli Zhang[ab], Xiaona Li[ab], Feiran Chen[ab], Xuesong Cao[ab], Chuanxi Wang[ab], Liya Jiao[ab], Le Yue[ab*], Zhenyu Wang[ab]

[a] *Institute of Environmental Processes and Pollution Control, School of Environment and Ecology, Jiangnan University, Wuxi 214122, China.*

[b] *Jiangsu Engineering Laboratory for Biomass Energy and Carbon Reduction Technology, Wuxi 214122, China.*

*Corresponding author:

Tel.: +86 0510 85911911

E-mail address: leyue@jiangnan.edu.cn (Dr. Le Yue)

Text S1 *HPLC-MS/MS operating parameters used in this study*

The components of theanine, catechins and alkaloids were detected by the high-performance liquid chromatography-tandem mass spectrometry (HPLC-MS/MS, Thermo Scientific, Germany). The fresh tea buds were ground into a powder. Then, samples were separated at 35 °C on a Thermo Scientific UPLC Vanquish equipped with a HSS T3 column applying the following gradient at a flow rate of 0.35 μL/min. The eluents in negative/positive mode were eluent A (0.01% v/v formic acid in water) and eluent B (0.01% formic acid in acetonitrile). The solvent gradient was set as follows: 5 % B, 0 min; 5 % B, 1 min; 95 % B, 11.0 min; 95 % B, 12min; 5% B, 12.1min; 5 % B, 14 min. The following dd-MS^2 product ion spectra were collected at resolution of 17500, isolation window of 3.0 m/z, and collision energy of nce: 15, 30, 40, respectively.

The metabolites in tea buds also were determined by HPLC-MS/MS. After the plant sample extraction, 10 μL samples were injected onto a Acquity HPLC HSS T3 (2.1×100 mm, 1.8 μm) using an 18-min linear gradient at a flow rate of 0.35 $mL \cdot min^{-1}$. The eluents in negative/positive mode were eluent A (0.1% v/v formic acid in water) and eluent B (0.1% formic acid in acetonitrile). The solvent gradient was set as follows: 5% B, 0 min; 5% B, 1.5 min; 100% B, 10.0 min; 100% B, 11 min; 100% B, 11.5min; 5% B, 14 min. Nitrogen was used as sheath gas (35 $L \cdot min^{-1}$) and aux gas (15 $L \cdot min^{-1}$). The spray voltage was set as 3 kV (-3 kV for negative mode and 3 kV for positive mode) and capillary temperature was 320 °C. The resolution of full MS was set as 70000 and scan range was acquired between 70 to 1050 m/z in full MS-dd MS^2 mode. The following dd-MS^2 product ion spectra were collected at resolution of 17500, isolation

window of 1.5 m/z, and collision energy of nce: 20, 40, 60. The raw data files were processed using Compound Discoverer 3.1 software coupled with the mzCloud and S-7 Chem Spider libraries.

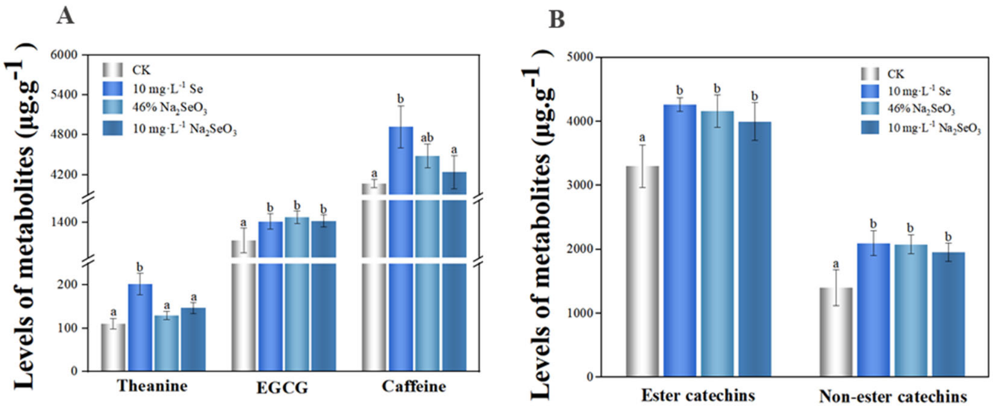

Figure S1. Changes in the content of (A) theanine, EGCG, caffeine and (B) esters, and non-ester catechins in FDDB treated with Se NMs and their equivalent ion control and fertilizer control. Letters following numbers indicate statistical significance - samples without common letters are significantly different at $p<0.05$ according to Duncan and Toucan tests.

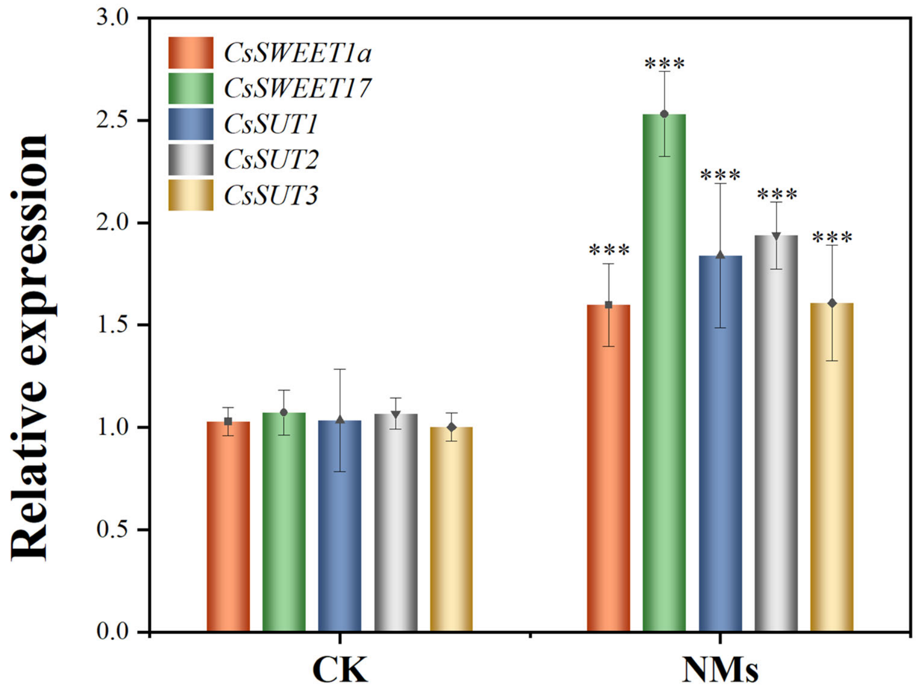

Figure S2. Relative expression of *CsSUT* and *CsSWEET* treated with Se NMs. The significant differences are marked with "***" ($p < 0.001$). The value represents the mean ± standard error (n = 5).

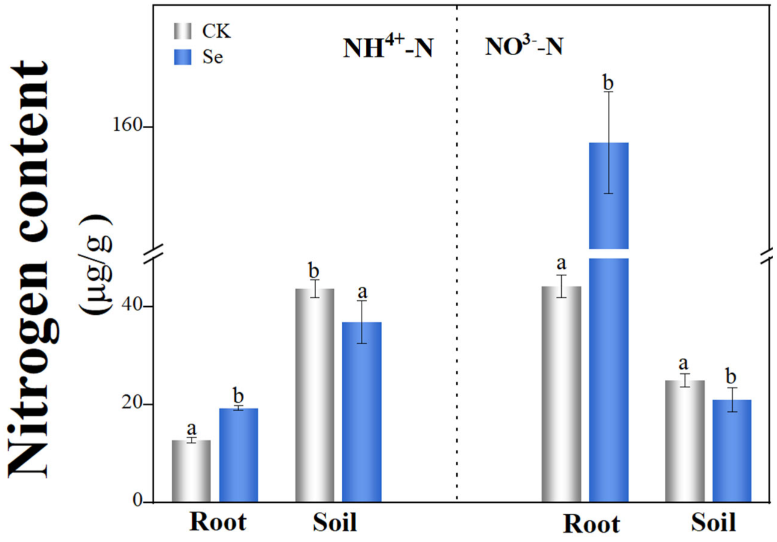

Figure S3. The content of ammonium and nitrate nitrogen in tea roots and rhizosphere soil under treatment with Se NMs. There was a significant difference between the treatment groups with different numbers ($p < 0.05$), the value represents the mean ± standard error (n = 5).

Table S1 Hydrodynamic diameter and Zeta potential of Se NMs.

Name	Value	Unit
Hydrodynamic diameter	156.8 ± 9.9	nm
Zeta potential	-24.0 ± 1.6	mV

Table S2 *Primer sequences of several genes related to metabolism and synthesis in tea*

Gene name	Forward Primer	Reverse Primer
GAPDH	TTGGCATCGTTGAGGGTCT	CAGTGGGAACACGGAAAGC
CsTs1	TCTTTCTGGACCTGTGAGTG	GCTTGAGGGTAGATAATGAGT
CsGs1	TAAAGTGCAACCGTCCCCTC	GTCCAAGCCAGTACCACCAA
CsGOGAT	CGAGGATCTTGCCCAACTGA	ACCCCAGAAGCAACAGTACC
CsPAL	GAATGCCGGTCTTATCCACT	CGGTGAACACCTTGTCAAAC
CsC4H	CGAGAGGTTCTTGGAAGAGG	AGAATTGGCAGAGCAAGGAT
CsCHI	CACAAAGAAGATTATGGGTGAAG	CAAACCTAGAAGTTGCCAAGAGT
CsANR	GAATGCCGGTCTTATCCACT	CGGTGAACACCTTGTCAAAC
CsLAR	GAATGCCGGTCTTATCCACT	CGGTGAACACCTTGTCAAAC
UGGT	GGCAAGAAGCTAATAGGGTCGTT	TTGTATCATTCGGAAGTGGTGGG
CsDFR	GTATTTGGACCGATGTGG	TTGTACTCCGGGTATTTC
CsANS	GGCCACAAGTGCCTACAATTG	CCCATGATTCACCAAATGCA
CsAPP1	GCTCAGTTGGGTTGGGTGGTTG	CGGCGAGGAGTGGAAGTGAA
CsAPP2	GCAGTGATTGGGTCAGGAGTGT	GCAGCGAGGAGAGTAGAAGTGT
CsAPP5	CTCTGTGGCTGCATGGGCTATG	TGGATGACTAGGCGGCATTGG
CsAPP6	TTGCGGTTGTCTTGGCTATGCT	CGGCTCTCTGCGAATGCGAATA
CsAPP8	CGGCACGGAATGGCTCTCAAT	TGGAGAACGGATAGGCGAAAGC
CsSWEET1a	AGCCAAGAAACCTCCTAATACC	TACTTGCTCGATCGCTTCTCTT
CsSWEET17	GGTTTCTCGGCAATACAGCATC	GGTGATCCTTGGTGCTTCCATT
CsSUT1	CATGCGATGCTAACTGTGCC	TTCAGCATGGCTCGACTGTT
CsSUT2	GGAATCGACTCCGATCTGCC	TGCAAAGCCCAACCGAATT
CsSUT3	TGTGGTCGGAGCTATTGCAG	CAGTGAGAAGGGGCAGAAGG
